# Mitochondria organize the cellular proteostatic response and promote cellular senescence

**DOI:** 10.15698/cst2019.04.181

**Published:** 2019-03-15

**Authors:** Yanjun Li, Lei Liu, Yushan Zhu, Quan Chen

**Affiliations:** 1 College of Life Sciences, Nankai University, Tianjin, China.; 2 State Key Laboratory of Membrane Biology, Institute of Zoology, Chinese Academy of Sciences, Beijing, China.

**Keywords:** mitochondrial quality control, proteostasis, cellular senescence

## Abstract

Mitochondria have relatively independent protein quality control systems, including their own chaperones for protein folding and AAA proteases for protein degradation. Accumulating evidence has shown that cytosolic proteins and disease-causing misfolded proteins can be translocated into mitochondria and then impinge upon their function. It is important to understand the interplay between cellular proteostasis and mitochondria, as impaired proteostasis and mitochondrial dysfunction are causally linked with aging and age-related disorders. This review highlights our recent finding showing that the outer mitochondrial membrane protein FUNDC1, a previously reported mitophagy receptor, interacts with the chaperone protein HSC70 to mediate the mitochondrial translocation of cytosolic proteasomal substrates via the TOM/TIM complex into the mitochondrial matrix where they can be degraded by LONP1 protease. Excessive accumulation of unfolded proteins within mitochondria triggers the formation of Mitochondrion-Associated Protein Aggregates (MAPAs), which are subsequently autophagically degraded in a FUNDC1-dependent manner. We suggest that mitochondria actively organize the cellular proteostatic response and that the interaction between FUNDC1 and HSC70 may represent a new link between impaired proteostasis, mitochondrial dysfunction and cellular aging.

Timely disposal and degradation of misfolded proteins and their aggregates is critical for cellular proteostasis and the well-being of a cell. During evolution, cells have acquired a sophisticated system to monitor protein quality and maintain protein homeostasis. This system consists of chaperones and two proteolytic pathways, the ubiquitin-proteasome pathway and the autophagy-lysosome pathway [1, 2]. Chaperones are able to recognize misfolded proteins to assist their refolding. If this fails, chaperones will facilitate the degradation of terminally misfolded proteins through the ubiquitin-proteasome system (UPS) [3]. Alternatively, chaperones may induce the misfolded proteins to form protein aggregates, which can be subsequently delivered to auto-lysosomes for degradation [4]. The proteasome and autophagy pathways share substrates, effectors and regulators, which allows cross-talk between them and compensation for one another in time of need [3]. One of the major functions of chaperones is to select the proteolytic pathway their client will follow, based on the other chaperones or co-chaperones they bind and the properties of their substrates [3]. The repertoire of chaperones includes members of the heat shock protein (HSP) families HSP100, HSP90, HSP70, HSP60, HSP40 and the small HSPs (sHSPs) [5]. HSP70-family proteins are the best characterized chaperones in cells to date, and HSP70 and HSC70 are two cytosolic members of this family which share 85% homology [6].

Mitochondria are multitasking cellular organelles that are essential for diverse cellular functions including energy production, redox signaling, immune responses and programmed cell death. Mitochondria have their own genome (mtDNA), which encodes less than 1% of the mitochondrial proteome [7]. To maintain their vital and diverse functions, mitochondria require the import of nuclear DNA-encoded proteins via the TOM/TIM complexes, coordinated expression and integration of nuclear DNA- and mtDNA-encoded OXPHOS subunits, and timely and extensive communication between nuclei and mitochondria [8]. Mitochondria have quality control systems that are relatively independent from the rest of the cell. They are well equipped with numerous chaperones, such as HSP60, HSP10, Mortalin, TRAP1 and CLPX, and AAA proteases, including LONP1, CLPP, OMI, AFG3L2, APG7 and YME1L1, to monitor, assemble or degrade excess or misfolded mitochondrial proteins [9]. The protease LONP1 is localized in the mitochondrial matrix and is able to degrade oxidized or misfolded proteins within this compartment [10]. Defective mitochondrial protein homeostasis activates the mitochondrial unfolded protein response (mtUPR), which activates nuclear transcription of the genes encoding the proteins required for degradation or refolding of misfolded proteins in mitochondria [11]. In *Caenorhabditis elegans*, nuclear translocation of a subset of transcription factors or chromosome modifiers, including ATFS-1, DVE-1, LIN-65 and MET-2, is required for transcriptional activation of the genes encoding mitochondrial chaperones or proteases [12, 13]. Interestingly, mitochondrial stress in neurons can evoke mtUPR in distal tissues in *C. elegans*, and this response is called cell non-autonomous mtUPR. Several “mitokines” including serotonin, the Wnt ligand EGL-20, and the neuropeptide FLP-2 have been reported to trigger such a response [14–16]. In mammalian cells, ATF5 is required for the activation of mtUPR in response to oxidative and mitochondrial proteotoxic stress [17]. The cytosolic UPS is also involved in the quality control of mitochondrial proteins, especially the mitochondrial precursor proteins and outer membrane proteins. Cdc48 (known as p97 in mammals), a core protein in ER-associated degradation (ERAD), is suggested to function in extracting damaged proteins from mitochondrial membranes and delivering them to the proteasome for degradation; this process is called mitochondrion-associated degradation (MAD) [18]. At the organellar level, damaged mitochondria can be segregated through mitochondrial fission, and subsequently degraded by mitochondrial autophagy, or mitophagy [19]. Accumulation of misfolded proteins at the site of mitochondria may trigger cell senescence or apoptosis (programmed cell death).

Several cytosolic E3 ligases play roles in the quality control of outer mitochondrial membrane (OMM) proteins and in mitophagy. The best characterized example is Parkin, an E3 ligase whose mutation causes juvenile Parkinson's diseases [20]. Parkin plays crucial roles in mitophagy and the degradation of both mitochondrial and cytosolic proteins. Parkin can ubiquitinate itself or other specific substrate proteins in the cytosol for subsequent proteasomal degradation [20]. Some disease-associated cytosolic proteins, including septin5, synphilin-1, alpha-synuclein, Drp1, p62 and cyclin E, have been identified as Parkin substrates [20–23]. Parkin can also form a complex with HSP70 and HSC70-Interacting Protein (CHIP), which can enhance Parkin-mediated ubiquitination of PAELR [24]. Upon cellular insult and mitochondrial depolarization, Parkin cooperates with PINK1 to mediate mitophagy. When mitochondria are depolarized, PINK1 fails to reach the inner mitochondrial membrane and accumulates on the OMM, leading to the phosphorylation of ubiquitin and Parkin. Phosphorylated Parkin is recruited to the OMM through binding to phospho-ubiquitin, which leads to the extensive ubiquitination of OMM proteins, such as MFN1/2, MIRO and VDAC1 (Voltage-dependent anion-selective channel 1), as well as mitochondrial fission and rupture of the outer membrane [25]. USP30, a mitochondrial deubiquitinase, counterbalances Parkin activity to prevent mitochondrial protein degradation and mitophagy [25]. PINK1 can directly recruit NDP52 and OPTN to mitochondria. These receptors are involved in the recruitment of the autophagy core components ULK1 (Unc-51-like kinase 1), DFCP1 (Double FYVE-containing protein 1) and WIPI1 (WD repeat domain phosphoinositide-interacting protein 1) toward mitochondria for mitophagy [26]. Some other ubiquitin E3 ligases have also been shown to function in mitochondrial quality control. A recent study in neurons suggested that CHIP is also recruited to mitochondria and is required for mitophagy in response to bioenergetic stress [27]. In addition, a genome-wide siRNA screen identified SMURF1 as another E3 ligase required for the autophagic degradation of damaged mitochondria in cultured cells and *in vivo* [28].

Several mitophagy receptors have also been identified to play roles in mediating mitophagy through their direct interaction with the autophagy machinery. These receptors contain the classic LIR motif that directly interacts with LC3 for the initiation of mitophagy. We have previously identified an OMM protein, FUNDC1, as a novel mitophagy receptor that functions under hypoxia [19]. In normoxic conditions, the Tyr-18 and Ser-13 residues of FUNDC1 are phosphorylated by SRC kinase and CK2 respectively, which prevents its binding to LC3., The activity of PGAM5, a mitochondrially localized phosphatase that can dephosphorylate FUNDC1 at Ser13, is inhibited through its interaction with BCl-xL [29]. When cells are challenged by hypoxia or other mitochondrial stresses, dephosphorylation of FUNDC1, likely due to the dissociation of the FUNDC1-SRC and FUNDC1-CK2 complexes and release of PGAM5 from rapidly inactivated Bcl-xL, enhances its interaction with LC3 to initiate mitophagy [29, 30]. Interestingly, a mitochondrially localized E3 ligase, can directly interact with FUNDC1 to mediate its ubiquitination and degradation, and thereupon tune down mitophagy at the early stage of mitochondrial stress [31].

We found that p62 is recruited to mitochondria when FUNDC1 is expressed, even in the absence of the FUNDC1 LIR motif that is required for mitophagy [32]. In an effort to understand the role of p62 in FUNDC1-mediated mitophagy, we uncovered that FUNDC1 interacts with HSC70 to actively promote protein translocation to the mitochondria. Under normal protein homeostatic conditions, cytosolic proteins can be eliminated by CHIP-dependent proteasomal degradation [32]. Under proteostatic stress conditions, excessively accumulated cytosolic proteasomal substrates can be imported into the mitochondrial matrix via the TOM/TIM complex. Some of these proteins can be degraded by the mitochondrial protease LONP1; the rest are concentrated in certain regions and then segregated from the mitochondrial network in a FIS1-dependent manner [32]. Interestingly, we observed that the FUNDC1-HSC70 interaction promoted the formation of Mitochondrion Associated Protein Aggregates (MAPAs). MAPAs are a new type of protein aggregate, distinct from aggresomes which are formed at the microtubule organizing center (MTOC) under the influence of HSP70 [32] (**Figure 1**). They are not localized at the MTOC and have their own properties. Specifically, MAPAs are membrane-enclosed and consist of ubiquitinated proteins, the ubiquitin binding protein p62 and mitochondrial proteins, including mitochondrial membrane proteins and unfolded proteins in the mitochondrial matrix [32]. FUNDC1 is also incorporated into MAPAs and mediates their autophagic degradation through its interaction with LC3 [32] (**Figure 1**). The recruitment of proteasomal clients to mitochondria and their accumulation within mitochondria are important for triggering the formation of MAPAs. HSP70 shares 85% homology with HSC70, but it has limited capacity to interact with FUNDC1, and it negatively regulates the mitochondrial translocation of proteasomal substrates and the formation of MAPAs [32]. Although the exact composition of MAPAs remains to be defined, our results uncovered an unexpected contributory role of mitochondria in maintaining overall proteostasis in cells, by employing their own AAA protease or mitophagy machinery. A previous study in yeast also suggests that cytosolic misfolding-prone proteins are imported into the mitochondrial matrix via the TOM-TIM complex and degraded by the mitochondrial protease PIM1 (yeast homolog of Lon protease) [33]. Additionally, a study in *Drosophila* showed that downregulation of Tom40 (translocase of outer membrane 40), a key subunit of the translocase of the OMM complex, resulted in the accumulation of ubiquitin-positive protein aggregates, probably due to disturbed fusion between autophagosomes and lysosomes [34]. These results are consistent with our findings in human cells, and suggest that mitochondria have a conserved organizational role in the cellular proteostasis network from yeast to human.

**Figure 1:**
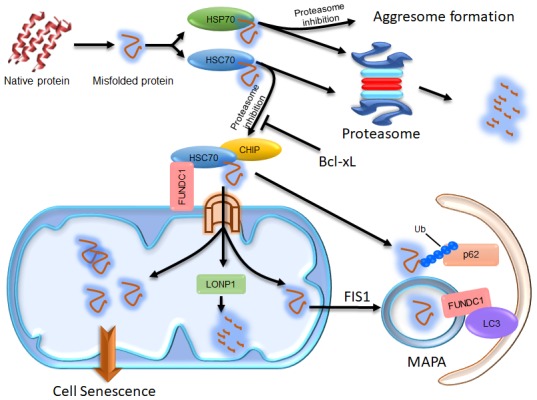
FIGURE 1: Illustration of the compensatory degradation of proteasomal substrates. The cytosolic chaperones HSP70 and HSC70 take their clients to the proteasome for degradation. If proteasomal activity is inhibited, HSP70 proteins take their clients to the microtubule organization center to form aggresomes, which may facilitate the autophagic degradation of the client proteins. HSC70 proteins, however, take their clients to mitochondria through the interaction of HSC70 with FUNDC1. Some of the client proteins are then imported into the mitochondrial matrix via the TOM/TIM complex and degraded by LONP1. The remains of the client proteins imported into the mitochondrial matrix may concentrate in certain region and become segregated from the mitochondrial network in a FIS-dependent manner to form MAPAs. The HSC70 clients at the surface of mitochondria can be ubiquitinated by the E3 ligase CHIP, which endows them with the ability to recruit p62 proteins. The ubiquitinated proteins and p62 at the surface of mitochondria and some of the mitochondrial membrane proteins, including FUNDC1, are all incorporated into the MAPAs. FUNDC1 can mediate recognition of the MAPAs by autophagosomes via its interaction with LC3. If the proteasomal client proteins imported into the mitochondrial matrix are not cleared in time, excessive accumulation of these proteins may disturb the mitochondrial function, consequently resulting in cellular senescence.

Loss of proteostasis and mitochondrial dysfunction are both causally linked with cellular senescence and aging [35]. Deposits of conformationally altered proteins cause cell morbidity in common human neurodegenerative pathologies and other aggregation-related diseases. Some of the disease-causing proteins which are prone to misfolding, such as alpha-synuclein, mutated mitochondrial HSP60, SOD and protease SPG7, and expanded polyQ Huntingtin aggregates, were found to be associated with impaired proteasomal activity and mitochondrial dysfunction [36, 37]. A wealth of reports have suggested that the proteasome contributes to the clearance of OMM proteins and mistargeted mitochondrial proteins [18], which may help to explain the correlation between impaired proteostasis and mitochondrial dysfunction. Our results suggest that when proteasomal function is inhibited, mitochondria are directly targeted through the interaction between FUNDC1 and HSC70 and are actively involved in handling the unfolded cytosolic proteins. Additionally, the cellular senescence induced by proteasomal inhibition is highly correlated with the mitochondrial accumulation of proteasomal substrates, as they are both positively regulated by FUNDC1 and HSC70, and negatively regulated by Bcl-xL, HSP70, LONP1, and FIS1 [32]. This suggests that mitochondrial accumulation of misfolded cytosolic proteins may act as a primary cause of the cellular senescence under conditions of proteotoxic stress or during aging. It is possible that in healthy cells, some of the unfolded cytosolic proteins are degraded by the proteasome while others are actively transported through the FUNDC1-HSC70 interaction into mitochondria where they are degraded by LONP1. In this scenario, only the age-related decline in the proteostasis network and mitochondrial quality control systems provokes the accumulation of misfolded proteins and mitochondrial dysfunction that lead to disease. It is also quite possible that some of the above-mentioned disease-causing proteins, such as mutant alpha-synuclein and polyQ Huntingtin proteins, are recruited to mitochondria through the FUNDC1-HSC70 interaction, and then result in mitochondrial damage, cell morbidity and diseases.

In sum, our study offers a mechanistic link between impaired proteostasis, mitochondrial dysfunction and cellular senescence. We further suggest an active role of mitochondria in proteostasis, including protein degradation, aggregate formation and auto(mito)phagic disposal of protein aggregates. Further work is underway to investigate the role of the FUNDC1-HSC70 interaction in MAPA formation and age-related aggregation diseases.
